# COVID-19 Outbreak Among Farmworkers — Okanogan County, Washington, May–August 2020

**DOI:** 10.15585/mmwr.mm7017a3

**Published:** 2021-04-30

**Authors:** James S. Miller, Michelle Holshue, Tia K.H. Dostal, Laura P. Newman, Scott Lindquist

**Affiliations:** ^1^Epidemic Intelligence Service, CDC; ^2^CDC COVID-19 Response Team; ^3^Washington State Department of Health.

Okanogan County, Washington, experienced increased community transmission of SARS-CoV-2, the virus that causes COVID-19, during summer 2020 (*1*). Multiple COVID-19 outbreaks occurred in agricultural settings, including a large outbreak among employees of a fruit grower during May–August. Because of this outbreak, Okanogan County Public Health and the Washington State Department of Health initiated one-time, on-site screening testing (*2*) of all orchard and warehouse employees in August 2020 and assessed risk factors for SARS-CoV-2 infection. Among 3,708 known orchard employees, a valid SARS-CoV-2 test result or information on COVID-19–like symptoms in the absence of a test was available for 3,013 (81%). Cumulative incidence of SARS-CoV-2 infection during approximately 3 months among tested orchard employees was 6%. Cumulative incidence was 12% in employees residing in the community, compared with 4% in employees residing in farmworker housing (p<0.001); point prevalence during the single screening testing event was 1% in both groups. Among 1,247 known warehouse employees, a valid result was available for 726 (58%). Cumulative incidence over approximately 3 months among tested warehouse employees was 23%, with substantial variation across job roles. Positive test results were received by 28% of employees who worked packing and sorting fruit, 24% of those in other roles in the packing and sorting area, 10% of forklift operators, 7% of employees in other warehouse roles, and 6% of office employees. Point prevalence among all warehouse workers was 1% at the screening testing event. Collaboration among employers, community groups, and public health authorities can reveal risk factors and help decrease farmworkers’ risk for SARS-CoV-2 infection in the community and the workplace. Creation of a COVID-19 assessment and control plan by agricultural employers, with particular focus on indoor workers whose jobs limit physical distancing, could reduce workplace transmission.

The Okanogan County fruit grower began referring symptomatic employees for SARS-CoV-2 testing in late May 2020. One-time SARS-CoV-2 screening testing of all employees was conducted on-site in late August.* Before then, asymptomatic employees were not systematically tested. Employees were eligible for inclusion in this investigation if they received at least one SARS-CoV-2 nucleic acid amplification test (NAAT) or antigen test with a positive or negative result, or if they were symptomatic but declined testing. A confirmed case was defined as the first positive SARS-CoV-2 NAAT or antigen test result received by an employee. A suspected case was defined as the presence of symptoms compatible with COVID-19 identified during work site symptom screening in an employee who declined testing.

Employees were classified by job site: orchard or warehouse. Orchard employees were further classified by housing location: congregate temporary farmworker housing (provided by the grower) or personally obtained housing in the community. All warehouse employees resided in the community. Warehouse employees were further classified into the following job roles: 1) sorting and packing fruit, 2) other roles supporting the fruit packing line, 3) forklift operation, 4) administrative (office setting), and 5) other warehouse roles (e.g., cleaning, maintenance, and transportation). Orchard employees worked predominantly outdoors. Warehouse employees generally worked indoors, although some warehouse roles involved some outdoor work. Warehouse employees performed similar work at three separate locations of differing size.

Descriptive analyses included cumulative incidence during approximately 3 months, stratified by housing category, job role, and work site. Chi-square tests and log-binomial regression models with robust error variance were used to evaluate differences in relative risk for SARS-CoV-2 infection across job roles and housing locations, with adjustment for work site among warehouse employees. Data were analyzed using Stata (version 15; StataCorp).^†^ This activity was reviewed by CDC and was conducted consistent with applicable federal law and CDC policy.^§^

During the 2020 harvest season, the fruit grower’s 4,955 employees included 3,708 orchard employees and 1,247 warehouse employees. Overall, 3,739 (75%) employees were included in this analysis, including 348 (9%) who received a positive SARS-CoV-2 test result (i.e., confirmed cases) and 71 (2%) suspected of having COVID-19. Among the 3,013 (81%) included orchard employees, 628 (21%) resided in the community and 2,385 (79%) in farmworker housing (Table 1). Among included orchard employees, 178 (6%) confirmed cases were identified, including 158 during symptomatic testing (May–August) and 20 during screening testing (August), along with 71 (2%) suspected cases. Among 196 symptomatic orchard employees tested, 158 (81%) received positive SARS-CoV-2 test results; 72 of 100 (72%) resided in the community, and 86 of 96 (90%) resided in farmworker housing. Over a period of approximately 3 months, the cumulative incidence of SARS-CoV-2 infection in orchard employees was 6%. Incidence was significantly higher among those residing in the community (12%) than among those residing in farmworker housing (4%) (p<0.001). Among orchard employees, the point prevalence during screening testing was similar across housing locations (1% in both groups; p = 0.950).

**TABLE 1:** SARS-CoV-2 test status and cumulative incidence of SARS-CoV-2 infection, by housing location among orchard employees at a fruit grower (N = 3,013) — Okanogan County, Washington, May–August 2020

**Measure**	**Residence no./total no. (%)**			**p-value**
	**Community housing**	**Farmworker housing**	**Total**	
**All testing**				
Total employees with positive SARS-CoV-2 test results	76/628 (12)	102/2,385 (4)	178/3,013 (6)	<0.001
Total employees with positive SARS-CoV-2 test results or suspected COVID-19	88/628 (14)	161/2,385 (7)	249/3,013 (8)	<0.001
**Symptomatic testing (May–August 2020)**				
Employees with positive test results during symptomatic testing (among all employees completing symptomatic testing)*	72/100 (72)	86/96 (90)	158/196 (81)	0.002
Employees with positive test results during symptomatic testing (among total included employees)	72/628 (11)	86/2,385 (4)	158/3,013 (5)	<0.001
Employees with suspected COVID-19^†^	12/628 (2)	59/2,385 (2)	71/3,013 (2)	0.408
**Screening testing (August 2020)**				
Employees with positive test results during screening testing^§^	4/552 (1)	16/2,287 (1)	20/2,839 (1)	0.950

* An additional 16 employees were recorded as having been tested during symptomatic testing but did not have a test result recorded and were not listed as having a suspected case of COVID-19. Among these 16 employees, 14 were tested during screening testing. The other two employees, who never had a test result recorded, were excluded from analysis. All 16 employees were excluded from the calculation of percentage of positive test results during symptomatic testing.

^†^ A suspected case was defined as the presence of symptoms compatible with COVID-19 in an employee who declined testing.

^§^ Employees who received negative test results during symptomatic testing or were considered to have suspected COVID-19 were tested during the screening testing. Employees who received positive test results during previous symptomatic testing were intended to be excluded from screening testing; however, five such employees were inadvertently retested and are excluded from this measure.

Among 726 (58%) included warehouse employees, 170 confirmed cases occurred, including 162 identified during symptomatic testing and eight during screening testing; no suspected cases were identified in these employees (Table 2). The percentage of tests that returned positive results during symptomatic testing could not be ascertained.^¶^ Cumulative SARS-CoV-2 incidence during approximately 3 months among warehouse employees was 23%, with substantial variation across job roles (ranging from 28% in employees packing and sorting fruit to 6% in office employees) and across work sites. Point prevalence during screening testing of warehouse workers was 1%. Information on employees’ use of face masks while working was not available.

**TABLE 2:** Characteristics, SARS-CoV-2 test status, and cumulative incidence of SARS-CoV-2 infection among warehouse employees (N = 726) at a fruit grower — Okanogan County, Washington, May–August 2020

**Measure**	**No./Total no. (%)**
**Symptomatic testing (May–August 2020)**	
Employees with positive test results during symptomatic testing (among total included employees)*	162/726 (22)
**Screening testing (August 2020)**	
Employees with positive test results during screening testing	8/548 (1)
**All testing**	
Total employees with positive SARS-CoV-2 test results^†^	170/726 (23)
Work site A	5/125 (4)
Work site B	44/118 (37)
Work site C	121/483 (25)
**All testing, by job role**	
Forklift operator	9/86 (10)
Packing and sorting fruit	84/304 (28)
Fruit packing support	30/126 (24)
Office	3/49 (6)
Other warehouse (e.g., maintenance, cleaning, transportation)	8/110 (7)
Unknown job role	36/51 (71)

* Full records of warehouse employees who received negative test results during symptomatic testing were not available, so the percentage of positive test results for symptomatic testing could not be determined.

^†^ Twelve new employees were tested at the screening testing event before starting work; they are excluded from analysis because they did not have any exposure to the work site before being tested. Seven employees had indeterminate results at the screening testing; five were retested and found to be negative, two were not retested and are excluded from analysis.

The first multivariate regression model used a binary outcome of confirmed SARS-CoV-2 infection among warehouse workers, with forklift operators and work site A as reference categories. The model identified a relative risk for infection of 2.7 for employees packing and sorting fruit (p = 0.002) and 2.4 for other packing roles (p = 0.015). The relative risk for office workers and other warehouse workers did not significantly differ from that of forklift operators (Table 3). The relative risk for infection was 6.8 (p<0.001) for employees at work site B and 5.8 for employees at work site C (p<0.001), compared with those at work site A. The second model examined SARS-CoV-2 infection in relation to job role and housing location for all employees. Results for warehouse job roles were similar, with significant associations between the packer and sorter role and other packing line roles and risk for SARS-CoV-2 infection. Orchard employees did not have a significant relative risk compared with forklift operators (relative risk = 1.2; p = 0.663). The relative risk for infection among those living in the community compared with those living in farmworker housing was 2.8 (p<0.001).

**TABLE 3:** Multivariate log-binomial regression models comparing risk for SARS-CoV-2 infection, by job role among employees at a fruit grower — Okanogan County, Washington, May–August 2020

**Measure**	**Relative risk (95% CI)**	**p-value**
Model for warehouse employees, assessing job role and work site*		
Forklift operator	Reference	—
Packing and sorting fruit	2.7 (1.4–5.2)	0.002
Fruit packing support	2.4 (1.2–4.7)	0.015
Office	0.6 (0.2–1.9)	0.347
Other warehouse (e.g., maintenance, cleaning, transportation)	0.8 (0.3–1.9)	0.552
Work site A	Reference	—
Work site B	6.8 (2.8–16.7)	<0.001
Work site C	5.8 (2.5–13.9)	<0.001
**Model for all employees, assessing job role and housing location^†^**		
Forklift operator	Reference	—
Packing and sorting fruit	2.6 (1.4–5.0)	0.003
Fruit packing support	2.3 (1.1–4.5)	0.020
Office	0.6 (0.2–2.1)	0.404
Other warehouse (e.g., maintenance, cleaning, transportation)	0.7 (0.3–1.7)	0.433
Orchard work	1.2 (0.6–2.2)	0.663
Lives in community	2.8 (2.1–3.8)	<0.001

**Abbreviation:** CI = confidence interval.

* Housing location was not included in this model because all warehouse workers resided in the community.

^†^ Work site was not included in this model because of collinearity for orchard workers.

## Discussion

Known risk factors for SARS-CoV-2 transmission and findings from previous outbreak investigations in other congregate housing and workplace settings suggest that farmworkers living in congregate housing and those working in larger groups indoors might be at elevated risk for SARS-CoV-2 infection (*3*–*5*). In other settings, farmworkers residing in the community were more likely to live in larger households with multiple adults working outside the home (*6*), which might also increase the risk for infection. In this investigation, cumulative incidence of SARS-CoV-2 infection was higher in orchard employees living in the community (12%) than among those residing in congregate temporary farmworker housing (4%). The point prevalence at the time of screening testing was equivalent in both groups. The difference in cumulative incidence could be explained by successful infection prevention efforts at farmworker housing facilities, differences in community exposures or behaviors between employees living in temporary farmworker housing and those living in the community, or more effective isolation of infected persons living in temporary farmworker housing. Alternatively, employees living in temporary farmworker housing might be less able or willing to seek SARS-CoV-2 testing. During the same period, cumulative incidence of SARS-CoV-2 infection in Okanogan County was approximately 2% (*1*). Incidence in both groups of orchard workers was higher than that in the overall community, although this comparison could be affected by differences in testing.

This investigation also demonstrated high cumulative incidence of SARS-CoV-2 infection among employees packing and sorting fruit or in other packing roles (24%–28%), who work primarily indoors in a large group, compared with that among forklift operators (10%), who work alone and partially outdoors, or among employees in other primarily indoor roles who tend to work alone or in small groups (6%–7%). Although this investigation could not directly assess transmission patterns, the significant differences in cumulative incidence of infection across job roles suggest that workplace transmission contributed to this outbreak. Differences in workplace prevention measures or differences in localized community transmission could explain the lower incidence at work site A, which is in a different town. Point prevalence among warehouse workers at the time of screening testing was 1%, which might reflect more widespread use of prevention measures, decreased community transmission, or decreased transmission as a result of the increased proportion of employees with immunity by that time. Early and improved access to testing for farmworkers and screening testing early in an outbreak might help to control transmission in future outbreaks. Focused efforts to maximize COVID-19 vaccination uptake among farmworkers also can help in preventing outbreaks, although such vaccines were not yet available at the time of this outbreak.

The findings in this report are subject to at least three limitations. First, the lack of individual exposure information, combined with a potentially high level of underascertainment of cases during symptomatic testing (i.e., cases in asymptomatic persons or persons who did not report their symptoms), might result in unmeasured confounding. Some employees might also have sought testing independently and not reported the results to their employer. Second, missing job role information for some employees could bias the comparison of cumulative incidence and regression models. Finally, the available employee records from the grower did not include employees’ race, ethnicity, preferred language, or other demographic information. Nationally, 83% of farmworkers identify as Hispanic (*7*). Hispanic or Latino, non-Hispanic Black, and non-Hispanic Asian/Pacific Islander farmworkers have been reported to experience increased incidence of COVID-19 (*8*). Collection of demographic information before or during an outbreak can help to identify potential exposures and disproportionately affected populations and guide prevention and messaging strategies.

Public health authorities and community organizations should prioritize culturally and linguistically tailored communication and interventions, including COVID-19 vaccination, to address farmworkers’ risk for acquiring COVID-19 in the community and in different work and living settings.** Creation of a COVID-19 assessment and control plan by agricultural employers, with particular focus on creating safer work environments for indoor workers whose job roles limit their ability to practice physical distancing, might help to reduce transmission in this group of disproportionately affected workers^††^ (*9*,*10*).

**Summary**

**What is already known about this topic?**

SARS-CoV-2 can spread rapidly in congregate housing and workplaces with limited physical distancing.

**What is added by this report?**

Among farmworkers employed by a fruit grower in Washington, SARS-CoV-2 incidence was higher among those living in the community (12%) than among those living in congregate temporary housing (4%). Incidence was higher among farmworkers packing and sorting fruit indoors (28%) than among those working alone or in small groups indoors or working outdoors (6%–10%).

**What are the implications for public health practice?**

Collaboration among employers, community groups, and public health authorities can help decrease farmworkers’ risk for COVID-19 in the community and the workplace, with particular focus on indoor workers whose jobs limit physical distancing.

## Footnotes

* Employees who previously received a positive test result were not retested during the screening testing. Although 16 tests had a reported test date of September 1, 2020, these tests were likely collected in late August at the screening testing event and occurred, or were reported to the fruit grower, on September 1.

† Section 27.9 of the Stata User’s Guide reviews the various approaches to generalized linear models available in Stata. https://www.stata.com/manuals/u.pdf

§ 45 C.F.R part 46, 21 C.F.R. part 56; 42 U.S.C. Sect. 241(d); 5 U.S.C. Sect. 552a; 44 U.S.C. Sect. 3501 et seq.

¶ For some warehouse employees tested during screening testing, records of previous negative tests during symptomatic testing were incomplete.

** Potential community interventions include 1) dedicated vaccination outreach efforts; 2) increased access to SARS-CoV-2 testing and, more broadly, to high quality and culturally competent health care; 3) improved housing access to decrease the risks for household transmission in crowded housing situations; and 4) policies that enable persons to isolate or quarantine if needed without fear of financial hardship or job loss.

†† Workplace prevention measures could include providing linguistically tailored education and training, promoting vaccination, cohorting employees, developing supportive policies for employees who need to isolate or quarantine, implementing engineering and administrative controls, and providing appropriate face masks or other personal protective equipment.

## References

[1] Washington State Department of Health. COVID-19 data dashboard. Olympia, WA: Washington State Department of Health; 2020. Accessed November 3, 2020. https://www.doh.wa.gov/Emergencies/COVID19/DataDashboard

[2] CDC. COVID-19: overview of testing for SARS-CoV-2 (COVID-19). Atlanta, GA: US Department of Health and Human Services, CDC; 2021. Accessed February 26, 2021. https://www.cdc.gov/coronavirus/2019-ncov/hcp/testing-overview.html

[3] Chew MH, Koh FH, Wu JT, Clinical assessment of COVID-19 outbreak among migrant workers residing in a large dormitory in Singapore. J Hosp Infect 2020;106:202–3. 10.1016/j.jhin.2020.05.034 32492454 PMC7261446

[4] Baggett TP, Keyes H, Sporn N, Gaeta JM. Prevalence of SARS-CoV-2 infection in residents of a large homeless shelter in Boston. JAMA 2020;323:2191–2. 10.1001/jama.2020.6887 32338732 PMC7186911

[5] Steinberg J, Kennedy ED, Basler C, COVID-19 outbreak among employees at a meat processing facility—South Dakota, March–April 2020. MMWR Morb Mortal Wkly Rep 2020;69:1015–9. 10.15585/mmwr.mm6931a2 32759914 PMC7454899

[6] Quandt SA, LaMonto NJ, Mora DC, Talton JW, Laurienti PJ, Arcury TA. COVID-19 pandemic among Latinx farmworker and nonfarmworker families in North Carolina: knowledge, risk perceptions, and preventive behaviors. Int J Environ Res Public Health 2020;17:5786. 10.3390/ijerph17165786 32785108 PMC7459606

[7] Hernandez T, Gabbard S. Findings from the National Agricultural Workers Survey (NAWS) 2015–2016: a demographic and employment profile of United States farmworkers. Rockville, MD: JBS International; 2018. https://www.dol.gov/sites/dolgov/files/ETA/naws/pdfs/NAWS_Research_Report_13.pdf

[8] Waltenburg MA, Rose CE, Victoroff T, ; CDC COVID-19 Emergency Response Team. Coronavirus disease among workers in food processing, food manufacturing, and agriculture workplaces. Emerg Infect Dis 2021;27:243–9. 10.3201/eid2701.203821 33075274 PMC7774547

[9] CDC. Agriculture workers and employers: interim guidance from CDC and the U.S. Department of Labor. Atlanta, GA: US Department of Health and Human Services, CDC; 2021. Accessed February 2, 2021. https://www.cdc.gov/coronavirus/2019-ncov/community/guidance-agricultural-workers.html

[10] CDC. Meat and poultry processing workers and employers: interim guidance from CDC and the Occupational Safety and Health Administration (OSHA). Atlanta, GA: US Department of Health and Human Services, CDC; 2021. Accessed February 2, 2021. https://www.cdc.gov/coronavirus/2019-ncov/community/organizations/meat-poultry-processing-workers-employers.html

